# Bioinformatics analysis and machine learning approach applied to the identification of novel key genes involved in non-alcoholic fatty liver disease

**DOI:** 10.1038/s41598-023-46711-x

**Published:** 2023-11-22

**Authors:** Elham Nazari, Ghazaleh Khalili-Tanha, Alireza Asadnia, Ghazaleh Pourali, Mina Maftooh, Majid Khazaei, Mohammadreza Nasiri, Seyed Mahdi Hassanian, Majid Ghayour-Mobarhan, Gordon A. Ferns, Mohammad Ali Kiani, Amir Avan

**Affiliations:** 1 Department of Health Information Technology and Management, School of Allied Medical Sciences, Shahid Beheshti University of Medical Sciences, Tehran, Iran; 2 Metabolic Syndrome Research Center, Mashhad University of Medical Sciences, Mashhad, Iran; 3 Medical Genetics Research Center, Mashhad University of Medical Sciences, Mashhad, Iran; 4 Recombinant Proteins Research Group, The Research Institute of Biotechnology, Ferdowsi University of Mashhad, Mashhad, Iran; 5 Basic Sciences Research Institute, Mashhad University of Medical Sciences, Mashhad, Iran; 6 Division of Medical Education, Brighton & Sussex Medical School, Falmer, Brighton, BN1 9PH Sussex UK; 7 Department of Pediatrics, Ghaem Hospital, Mashhad University of Medical Sciences, Mashhad, Iran; 8 College of Medicine, University of Warith Al-Anbiyaa, Karbala, Iraq; 9 Faculty of Health, School of Biomedical Sciences, Queensland University of Technology (QUT), Brisbane, 4000 Australia

**Keywords:** Computational biology and bioinformatics, Biomarkers

## Abstract

Non-alcoholic fatty liver disease (NAFLD) comprises a range of chronic liver diseases that result from the accumulation of excess triglycerides in the liver, and which, in its early phases, is categorized NAFLD, or hepato-steatosis with pure fatty liver. The mortality rate of non-alcoholic steatohepatitis (NASH) is more than NAFLD; therefore, diagnosing the disease in its early stages may decrease liver damage and increase the survival rate. In the current study, we screened the gene expression data of NAFLD patients and control samples from the public dataset GEO to detect DEGs. Then, the correlation betweenbetween the top selected DEGs and clinical data was evaluated. In the present study, two GEO datasets (GSE48452, GSE126848) were downloaded. The dysregulated expressed genes (DEGs) were identified by machine learning methods (Penalize regression models). Then, the shared DEGs between the two training datasets were validated using validation datasets. ROC-curve analysis was used to identify diagnostic markers. R software analyzed the interactions between DEGs, clinical data, and fatty liver. Ten novel genes, including *ABCF1, SART3, APC5, NONO, KAT7, ZPR1, RABGAP1, SLC7A8, SPAG9,* and *KAT6A* were found to have a differential expression between NAFLD and healthy individuals. Based on validation results and ROC analysis, *NR4A2* and *IGFBP1b* were identified as diagnostic markers. These key genes may be predictive markers for the development of fatty liver. It is recommended that these key genes are assessed further as possible predictive markers during the development of fatty liver.

## Introduction

Non-alcoholic fatty liver disease (NAFLD) is a range of chronic liver diseases resulting from the accumulation of excess triglycerides in the liver^1^. NAFLD affects about 25–30% of the population globally and is associated with an increased risk of other diseases, including type 2 diabetes mellitus, atherosclerotic cardiovascular disease, and chronic kidney disease (CKD)^2–5^. The frequency of NAFLD is anticipated to increase from 83 million in 2015 to 100 million by 2030^6^. The main reasons are an unhealthy diet and a sedentary lifestyle^7^. The advanced type of fatty liver disease is non-alcoholic steatohepatitis^8^, with features that include: fibrosis, necrotic inflammation leading to cirrhosis, and hepatocellular carcinoma^1,7,9,10^. The morbidity and mortality rates of cirrhosis and hepatocellular carcinoma are much greater than NAFLD^11^. Therefore, diagnosing and treating disease in the early stages might reduce the risk of liver damage and increase the survival rate^12,13^.

The gold standard for detecting NASH is liver biopsy, an invasive approach^14^. The other diagnostic tools ,resonance imaging^15^, and computed tomography (CT) whichare high-cost and time-consuming methods that burden the healthcare system financially^16,17^. Additionally, recognizing the higher level of lipid content and inflammatory factors such as C-reactive protein (CRP), IL-6, IL-18, IL-1b, IL-8, and TNF-a signify chronic inflammation in NASH development^18,19^. Therefore low-cost, reliable, and non-invasive methods are required to identify specific diagnostic biomarkers in the early stage of NAFLD.

In the progression of NAFLD, the molecular pathways are altered, leading to differential expression genes (DEGs). In the new era of technology, Machine learning (ML) is a novel artificial intelligence that has been widely performed to screen DEGs in different diseases and discover new diagnostic and prognostic biomarkers. Artificial intelligence enables processing data sets using programmed algorithms in logical models for performance tasks^20–22^. ML has various advantages, including automation, Handling multi-dimensional data, nonlinearity, low fault, and wide applications^23,24^.

In the current investigation, we screened the gene expression data of NAFLD patients and control samples from the public dataset GEO (Gene Expression Omnibus) to detect DEGs. Then, the correlation between the top selected DEGs and clinical data was evaluated.

## Methods and materials

### Workflow

The RNASeq data of fatty liver patients and clinical features were downloaded from the GEO dataset (GSE126848 and GSE48452). Filtering and normalization were performed as preprocessing, and the data quality was controlled using Principal Component Analysis (PCA). Before classification, feature selection was implemented using Relief-based algorithms to calculate the higher score for each feature. Then Penalize machine learning technique was used to detect the most important biomarkers. Eventually, the candidate genes were validated by other datasets.

### Data source

In the present study, two datasets from GEO were downloaded. The first dataset included 33,297 array-based expression profiling of 73 samples which were grouped into C (control = 14), O (obese = 27), S (steatosis = 14), and N (NASH = 18), and the second dataset consisted of 19786 gene expression from normal individuals (n = 14), obese (n = 12), NAFLD (n = 15) and NASH (n = 16) patients. The two datasets were extracted from https://www.ncbi.nlm.nih.gov/geo/geo2r/?acc=GSE48452 and https://www.ncbi.nlm.nih.gov/geo/query/acc.cgi?acc=GSE126848, respectively. The two datasets also have clinical and demographic variables considered in the analysis.

### Differential expression analysis (preprocessing)

Gene expression data were screened by filtering, and the zero expressions were eliminated; then, data were normalized with limma in R 4.1 software. The adjusted p < 0.05 and − 1.5 <|Log2FC (fold change) |< 1.5 were identified for subsequent analysis as significant genes. After that, Principal Component Analysis (PCA) which is a statistical procedure for visualizing whether the sample groups (control and patients) were separable and correlated was applied..

### Identifying Important genes and correlation between clinical/demographic factors with fatty liver

The effect coefficient of all factors on the fatty liver was calculated using Regularization regressions (LASSO, () Ridge, and Elastic Net) models. These models will be described as follows. Before the modeling, Relief-based feature selection was implemented. Weight by Relief is applied to calculate the weights of the attributes in the polynomial dataset. Chi-square and One way-ANOVA also were used to evaluate the relationship between clinical variables and disease, and Kolmogorov–Smirnov was used for normality test distribution. The binary correlation of some variables was examined using a correlation matrix. R4.1.and EVIEWS12 software was utilized for analysis.

### Regularization regression

In statistics and machine learning, Regularization regression is a type of regression analysis for variable selection and is used when train and test data are varying. To better manage many parameters or Multicollinearity between variables and reduce complexity, a “penalty” is added to cost function (Regularization) for the best fitting of training data. This reduce the variance of the test data, prevent over-fitting and enhance the prediction accuracy. Here are briefly introduced three Regularization regressions methods.

### Least Absolute Shrinkage and Selection Operator (LASSO) regression

The term Lasso stands for “least absolute shrinkage and selection operator”. Lasso uses shrinkage by shrinking data values to a central point such as mean. In this model, the regularization method is based on the absolute value of loss function. As a result, the target function in "Lasso Regression" is written as follows

$$\mathop{\varvec{\sum}}\limits_{{\varvec{i = 1}}}^{{\varvec{n}}} { = }\left( {{\varvec{yi}} -\varvec{\beta 0} - \mathop{\varvec{\sum }}\limits_{{\varvec{j = 1}}}^{{\varvec{p}}}\varvec{xij\beta j}} \right)^{{\varvec{2}}}\varvec{ + \lambda \sum j}\left| {\varvec{\beta j}} \right|$$

### Ridge regression

In the Ridge regression, the Quadratic Loss Function is used. In such way, the amount of penalty is determined as the sum of squares of coefficients.

Thus, if we consider the regression model as follows:

$${\varvec{y}}_{{\varvec{i}}}={\varvec{ \beta} }_{{\varvec{0}}} { + {\varvec \beta} }_{{\varvec{1}}} {\varvec{xi}}_{{\varvec{1}}} { +{\varvec L }+ {\varvec \beta} }_{{\varvec{p}}} {\varvec{x}}_{{{\varvec{ip}}}} { +{\varvec \varepsilon i, \quad i }= 1,} \ldots, {\varvec n}$$

The Ridge regression model is performed by minimizing the following function.

$$\varvec{Argmin ||y} -\varvec{\hat{y}||}_{{\varvec{2}}}^{{\varvec{2}}}\varvec{ = argmin\sum }\left[ {\varvec{yi{-} (\beta 0 + \beta 1x1 + \beta 2x2 + L + \beta pxp)}} \right]^{{\varvec{2}}} x$$

Note that argmin refers to values of ββ that minimize the desired function.

To estimate the regression parameters in the Ridge method, there is a constraint on the parameters which is written as follows.

$${\varvec{\beta}}_{{\varvec{0}}}^{{\varvec{2}}}\varvec{ + \beta }_{{\varvec{1}}}^{{\varvec{2}}}\varvec{ + } \cdots\varvec{ + \beta }_{{\varvec{p}}}^{{\varvec{2}}}\varvec{ \le C}^{{\varvec{2}}}$$

This constraint specifies that the sum of the squares of the parameters must be less than a constant or threshold value. In this way, the method of estimating the parameters will be as follows. It is clear that a balance is established between the existence of ββ parameters and their zeroing in the constraint section, and the num ber of related parameters and variables is optimized.

$${\mathbf{Argmin}} \, \left| {\left| { \, {\mathbf{y}}{-}{\mathbf{X\beta }}} \right|} \right| _{2}^{2} + {{\varvec{\uplambda}}} \, \left| {\left| {{\varvec{\upbeta}}} \right|} \right|_{2}^{2}$$

The λλ parameter here is called the Penalty Regulation (Regularization Parameter).

Note that regularization is done only for parameters β1β1 to βnβn, and intercept of β0β0 is an exception in this regard. Estimation of the parameters of the Ridge regression model according to the mentioned constraint will be as follows.

$$Bridge = \left( {\varvec{X}^{\varvec{T}} X + \varvec{\lambda I}} \right)^{{ - 1}} \varvec{X}^{\varvec{T}} \varvec{y\beta}{}^{\wedge}$$

### Elastic Net regression

Elastic Net Regression, by combining lasso regression and Ridge regression, overcomes their disadvantages and is a reliable alternative to them. Thus, if you are faced with a model whose descriptive variables are correlated with each other, it is better to use Elastic Net regression. In this method, Loss Function and Quadratic Loss Function are applied to the model simultaneously. As a result, the target function in the elastic network regression will be written as follows.

$${\varvec{min}}\left( {\sum \in^{{\varvec{2}}}\varvec{ + \lambda 1}\sum\varvec{\beta i + \lambda 2}\sum\varvec{|\beta i|}} \right)$$

Considering the multiple linear regression model, it can also be written as follows.

$$min\left( {\varvec{\sum yi{-}}\left( {\varvec{\beta 0 + \beta 1X1 + \beta 2X2 + } \cdots\varvec{ + \beta kXk}} \right)\varvec{2 + \lambda 1\sum \beta 2i + \lambda 2\sum |\beta i|}} \right)$$

Note that, like the lasso regression and the Ridge regression, in the Elastic Net regression there is no assumption that the residual is normal. Also, the intercept is not involved in the regularization^25,26^.

### Protein–protein interaction network

The online string tool (https://www.string-db.org/) was performed to analyze DEGs’ protein–protein interaction with a score of 0.4. Moreover, all the networks were depicted using R software.

### GO pathway analysis

The enrichment GO analyses were performed to detect the molecular function of DEGs in NAFLD using Go package, nrichGO, and gseGO package.

### Validation of biomarkers gene expression

The expression levels of candidate genes in patients were verified by using Gene Expression Omnibus (GEO) dataset (GSE89632 and GSE63067). The validation datasets consisted of data from patients with fatty liver, which were downloaded from this web tool, and the pre-processing was performed.

### Combine ROC curve

The receiver operating characteristic (ROC) curve was performed to evaluate the efficacy of the diagnostic model. Specificity, sensitivity, area under the ROC curve, positive predictive value, negative predictive value, and cut-off value were assessed for each gene and their combination. All the procedures were analyzed by package combioROC in R.

## Results

### Data description

**Figure 1:**
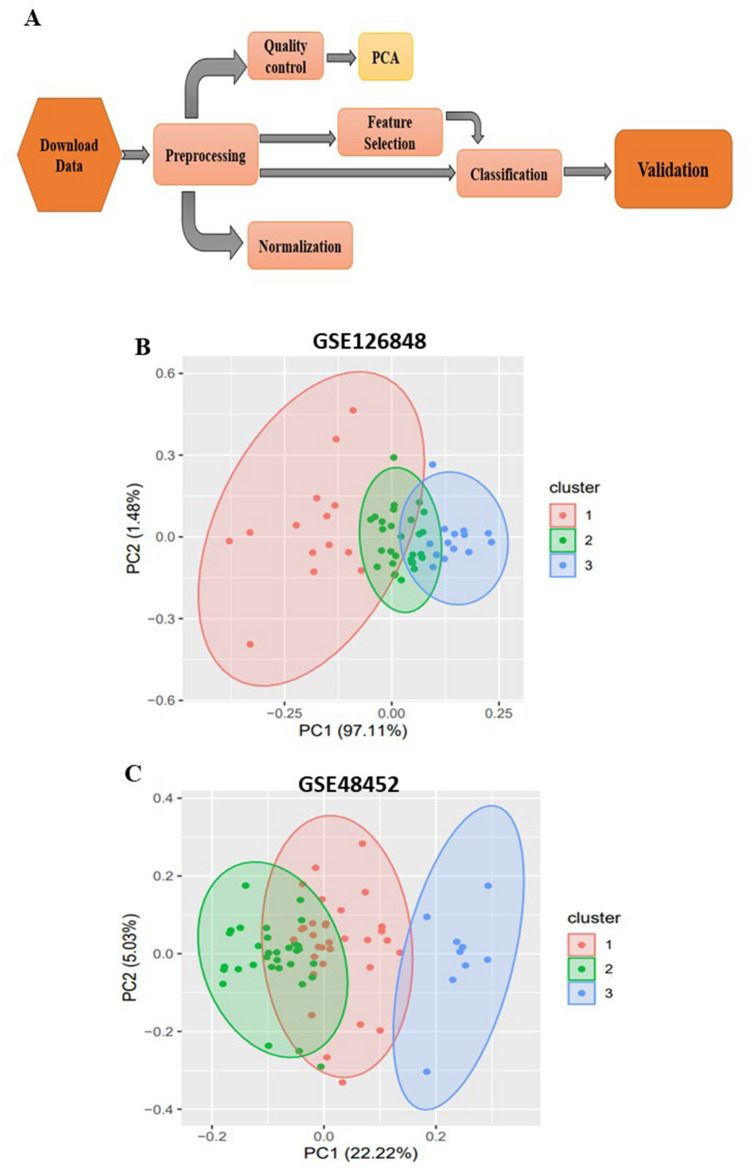
(**A**) The flow of work; The result of Principal Component Analysis (PCA) indicated the discrimination between patients and healthy samples in (**B**) GSE126848, and (**C**) GSE48452 datasets.

Figure 1A shows the overall workflow. Tables 1 and 2 show the mean and standard deviation of the quantitative variables. The frequency and percentage of attributes in the study are also mentioned. The result of PCA indicated the discrimination between patients and healthy samples (Fig. 1B and C).

**Table 1:** The clinical characteristics of datasets.

Attributes	Sub category	Frequency (%)
GSE48452		
Case–control	Control	14 (19.2)
	Obese	27 (37)
	NAFLD	14 (19.2)
	NASH	18 (24.7)
Gender	Male	15 (20.5)
	Female	58 (79.5)
		SD ± Mean
Fat		25.45 ± 3.77
Inflammation		0.48 ± 0.096
Age		45.92 ± 1.32
BMI		40.45 ± 1.38
Nas		1.64 ± 0.252
Fibrosis		0.479 ± 0.1
Lar		4.32 ± 0.57
Leptin		25.44 ± 2.62
GSE126848		
Case–control	Control	14(24.6)
	Obese	12 (21.1)
	NAFLD	15 (26.3)
	NASH	16 (28.1)
Gender	Male	47 (82.5)
	Female	10(17.5)

The ensemble ID was converted to gene name by Biotool.fr and the ID ref of GEO was converted by g: profiler. All the none genes were deleted from the study.

**Table 2:** Association between Clinical/Demographic factors and fatty liver.

Dataset name	Variable1	Variable2	Result
GSE48452	Response variable	Fat	F = 134.283 sig = 0.00 DF = 72 Sum of square = 74,782.082
		Inflammation	F = 53.514 Sig = 0 Df = 72 Sum of square = 48.219
		BMI	Sig = 0 F = 2.374 Df = 72 Sum = 9979.798
		Fibrosis	Sig = 0 Df = 72 F = 12.224 Sum = 52.719
		Nas	Sig = 0 F = 248.986 Df = 72 Sum of square = 332.760
		Leptin	Sig = 0 F = 6.037 Df = 72 Sum of square = 36,056.84
		Lar	Sig = 0 F = 5.771 Df = 72 Sum of square = 1715.555
GSE126848	Response variable	Sex	Sig = 0 Df = 3 Pearson chi-square = 11.376

### Weight by Relief

**Figure 2:**
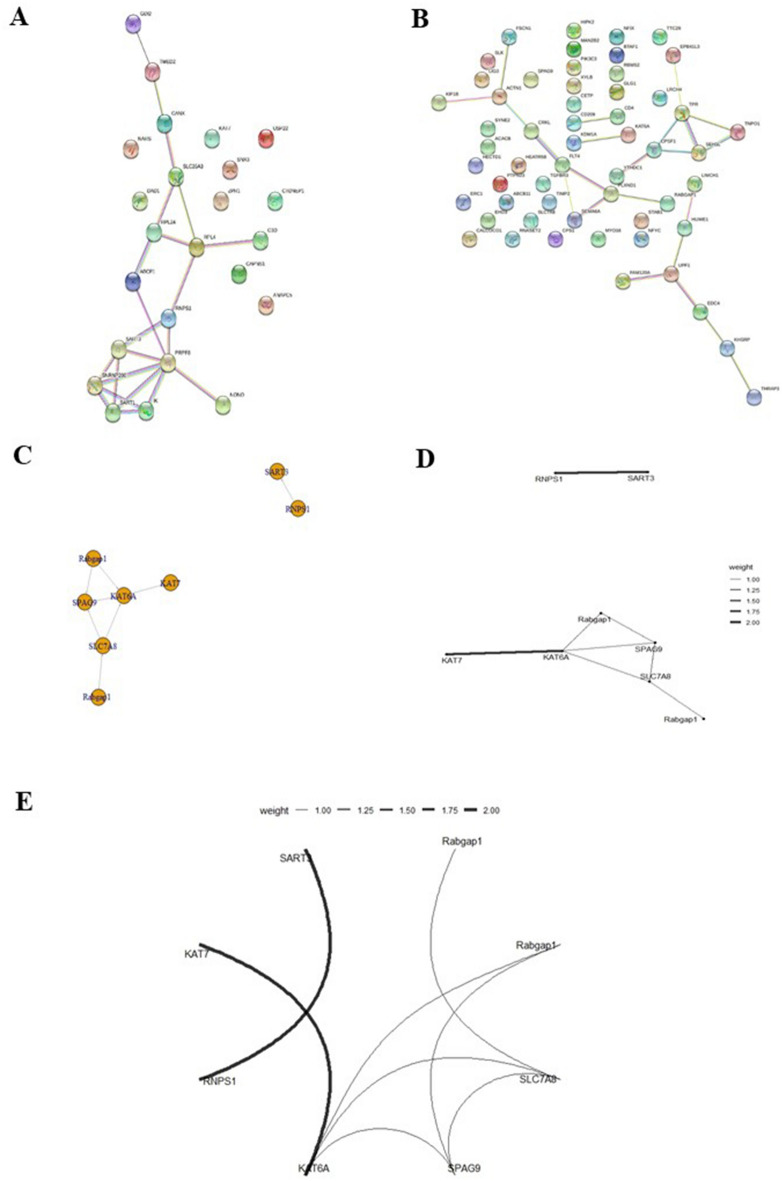
Protein–protein interaction (PPI) network of differentialy expressed genes (DEGs); (**A**) PPI in GSE48452 dataset, (**B**) PPI in GSE126848 dataset, (**C**–**E**) PPI between key genes.

The weight of the variables of the two datasets can be seen in Fig. 2. The data show a significant correlation between DEGs and fatty liver.

### Comparison of three methods for identifying important coefficients (GSE126848)

Three methods of Regularization regression, including LASSO, Ridge, and Elastic Net, were candidate to identify the effect coefficient of variables on fatty liver. Each of the color lines belongs to the coefficient of one variable, which with increasing Lambda parameter, the number of non-zero coefficients decreases, and the size of the coefficients becomes smaller and approaches zero. After fitting the model, with five k-fold cross-validation, the optimal value of the Lambda parameter was determined, and the results of the final model were reported. The model’s cross-validation results were plotted in a graph containing different values of Lambda versus Train/Test error, which shows the Train/Test Error related fitted models in different Lambda sizes (Fig. S1). Among the three implemented methods with five k-fold cross-validation for evaluation, the Elastic Net method had the highest performance (Lambda at minimum error: 11.87, R^2^ = 0.999 and alpha = 0.5, l1 Norm = 1.31). The area under the curve was approximately 0.99 with a confidence interval (0.95,1). The Elastic Net is an extension of the lasso robust to extreme correlations among the predictors. The results of Elastic Net method for identifying important factors can be seen in Table 3.

**Table 3:** The most important genes coefficients on the fatty liver) GSE126848 and GSE48452).

GSE126848		
Gene name	Coefficient	Full name
*RABGAP1*	4.2404	RAB GTPase Activating Protein 1
*SLC7A8*	3.4923	Solute Carrier Family 7 Member 8)
*SPAG9*	2.3551	Sperm Associated Antigen 9
*KAT6A*	1.7418	Lysine Acetyltransferase 6A

GSE48452		
Gene name	Coefficient	Full name
*ABCF1*	8.3581	ATP Binding Cassette Subfamily F Member 1
*SART3*	7.891196	Spliceosome Associated Factor 3
*RNPS1*	6.815792	RNA-binding protein with serine-rich domain 1
*ANAPC5*	3.360324	Anaphase Promoting Complex Subunit 5
*NONO*	2.726974	Non-POU Domain Containing Octamer Binding
*CTDNEP1*	1.722116	CTD Nuclear Envelope Phosphatase 1
*KAT7*	0.855729	Lysine Acetyltransferase 7
*ZPR1*	0.805633	ZPR1 Zinc Finger

### Comparison of three methods for identifying important coefficients (GSE48452)

The three methods of Regularization regression were used to identify candidate genes that may be used to identify the effect coefficient of variables on fatty liver. Each of the colored lines represents the coefficient of one variable, which with increasing Lambda parameter, the number of non-zero coefficients decreases, and the size of the coefficients becomes smaller and approaches zero. After fitting the model, with five k-fold cross-validation, the optimal value of Lambda parameter was gained, and the results of the final model were reported. The results of cross-validation of the model were plotted in a graph containing different values of Lambda versus Train/Test error, which shows the Train/Test Error related fitted models in different Lambda sizes (Fig. S2). Among the three implemented methods with five k-fold cross-validation for evaluation, the Elastic Net method had the highest performance (Lambda at minimum error: 0.00, R^2^ = 0.999 and alpha = 0.5, l1 Norm = 213.66). The area under the curve was approximately 0.99 with a confidence interval (0.95, 1). The Elastic Net is an extension of the lasso robust to extreme correlations among the predictors. The results of Elastic Net method for identifying important factors can be seen in Table 3.

### Comparison of three methods for identifying common genes between two datasets

After normalization with significant p-value and log fold change, the common genes between GSE126848 and GSE48452 were 155, which were used to identify the most important candidate genes using Lasso Machine Learning technique. For GSE126848 dataset with 57 samples, among the three implemented methods with five k-fold cross validation for evaluation, the Lasso method had the highest performance (Lambda at minimum error: 1.451, R^2^ = 0.999 and alpha = 1, L1 Norm = 15.96)(Fig. S3). For GSE48452 dataset with 73 samples, among the three implemented methods with five k-fold cross-validation for evaluation, the Lasso method had the highest performance (Lambda at minimum error: 0.01388, R^2^ = 0.999 and alpha = 1, L1 Norm = 15.96) (Fig. S4).

### Identification of dysregulate expression genes (DEGs)

The GSE48452 chip contained 14 NAFLD, 18 NASH, and 27 obese samples, among which 15,000 genes and 1400 DEGs were identified. Moreover, the GSE126848 chip had 15 NAFLD, 16 NASH, and 12 obese 9540 genes, and 843 DEGs were found in this dataset based on specific criteria (Table 2). Furthermore, the commonality of novel genes between two datasets was assessed after normalization. Then Penalize machine learning technique was used to detect the most important common genes between two data sets. The results indicated that eighty-eight genes were common between two datasets (Table 3).

### PPI network construction

**Figure 3:**
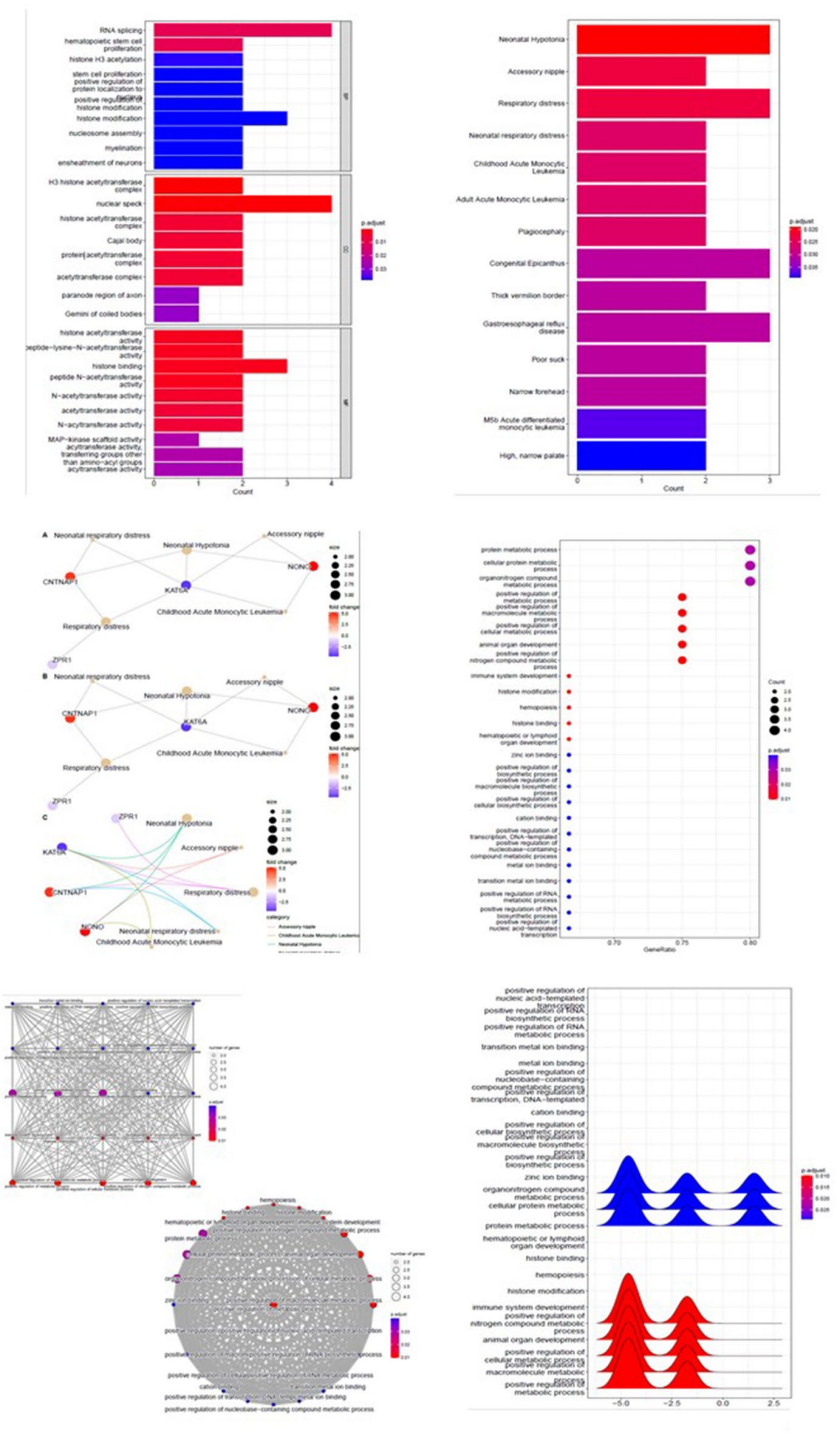
Gene ontology (GO) functional annotation of top DEGs enrichment terms in fatty liver disease; molecular function (MF) of DEGs was mainly enriched in histone acetyltransferase activity, peptide − lysine − N − acetyltransferase activity, histone binding, and peptide *N* − acetyltransferase activity. The biological process (BP) consisted of RNA splicing, hematopoietic stem cell proliferation, and histone H3 acetylation. The cell component (CC) was detected in nuclear speck and H3 histone acetyltransferase complexes.

As seen in Fig. 3, the PPI interaction network of DEGs was analyzed and depicted by String, and the interaction score was set at 0.4. As we can see in the network analysis, the *KATA6A* and *KAT7* genes were strongly correlated, as well as, a significant correlation was detected between the *SART3* and *RNPS1* genes.

### Gene ontology analyses of DEGs

R software results showed that the molecular function of DEGs was enriched in histone acetyltransferase activity, peptide–lysine–*N*–acetyltransferase activity, histone binding, and peptide *N*-acetyltransferase activity. The biological process includes RNA splicing, hematopoietic stem cell proliferation, and histone H3 acetylation. Furthermore, the cell component was detected in nuclear speck and H3 histone acetyltransferase complexes (Fig. 3).

### Validation using validation datasets

The five common genes between two datasets, GEO126848 and GEO48452, were validated by two other datasets, consisting of GSE89632 and GSE63067. The results indicated the five most important novel genes in fatty liver, including NR4A2, ZEB2, IGFBP1b, AKR1B10, DHRS2, and UGT2B17 (Table 4).

**Table 4:** Common genes between GSE126848 And GSE48452 validated in other datasets.

Gene name	Full name
AKR1B10	Aldo–keto reductase family 1 member B10
DHRS2	Dehydrogenase/reductase SDR family member 2
UGT2B17	UDP-glucuronosyltransferase 2B17
IGFBP1	Insulin-like growth factor-binding protein 1
NR4A2	Nuclear receptor subfamily 4 group A member 2
ZNF653	Zinc finger protein 653
ZEB2	Zinc finger E-box-binding homeobox 2

### GO pathway analyses

Enrichment analysis results showed that the molecular function of shared DEGs was mainly enriched in structural molecule activity. The biological processes were peptide biosynthetic process and translation. Moreover, the main involved cell components were ribonucleoprotein complex and ribosome. Reactom pathway analysis revealed that metabolism of RNA and cellular responses to stress and stimuli were the most significant dysregulated pathways in fatty liver (Fig. 3).

### ROC curve for identification of diagnostic markers

Our finding showed that *NR4A2* alone (AUC of 0.92, 95% CI with a sensitivity of 1.00and specificity of 0.71), and also, its combination with ZEB2 (AUC of 0.92, 95% CI with a sensitivity of 0.90 and specificity of 0.85) had the highest rank of ROC analysis and can be considered as diagnostic markers (Fig. S5 and Table S1). Moreover, our data revealed that *IGFBP1b* alone (AUC of 0.90, 95% CI with a sensitivity of 0.89 and specificity of 0.87), and its combination with AKR1B10, DHRS2, IGFBP1, and UGT2B17 with AUC of 0.96, 95% CI with a sensitivity of 0.94 and specificity of 0.95, also had the highest rank (Fig. S6 and Table S2).

### Association between Clinical/Demographic factors and fatty liver

A significant relationship was obtained between fat, fibrosis, BMI, inflammation, and fatty liver.

### Investigation of the binary correlations of Clinical/Demographic influence variables on fatty liver

**Figure 4:**
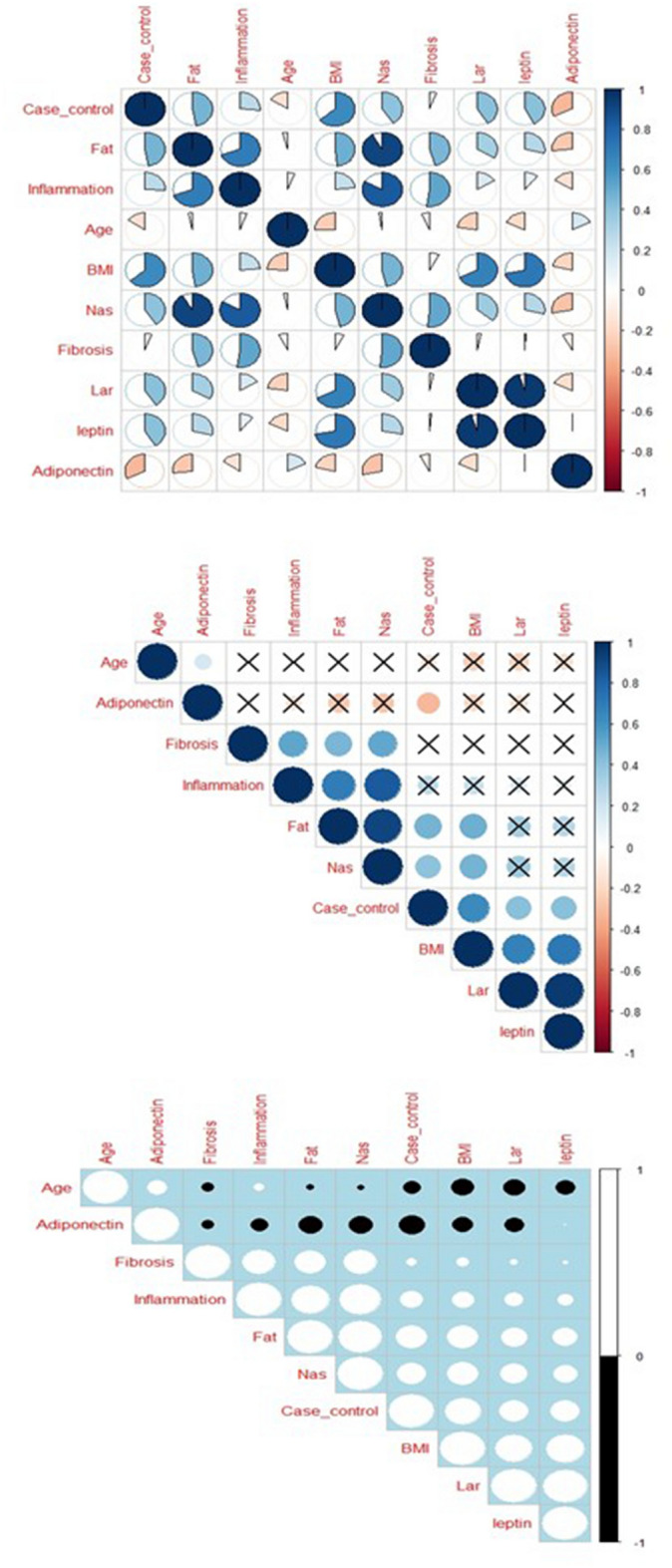
Correlation matrix for showing significant relationship between clinical/demographic influence variables in fatty liver disease.

Using the correlation matrix, we examined the correlation between pairs of variables. The results are shown in Fig. 4. Note that a correlation coefficient of less than 0.3 is considered weak, the coefficient between 0.3 and 0.6 is moderate, and a coefficient greater than 0.6 is considered strong. Coefficients with a P-value less than 0.05 are also significant. As we concluded from Fig. 4, BMI, Lar, Leptin, Fat, and Nas have correlated significantly with the disease in positive direct and Adiponectin correlated with fatty liver negatively.

## Discussion

For the first time in the present study, we have used machine learning approaches to compare the gene expression profile of individuals with NAFLD, NASH, and obesity with healthy individuals.

Firstly, we analyzed GSE126848 and GSE48452 datasets separately, and the results detected 9540 and 1400 DEGs genes in the two datasets, respectively. We reported genes with higher coefficients in each dataset. Six genes, including *ABCF1, SART3, APC5, NONO, KAT7, and ZPR1* were identified in GSE48452 datasets, as well as four genes, including *RABGAP1, SLC7A8, SPAG9*, and *KAT6A* were detected in GSE126848 dataset with a different expression between NAFLD and healthy samples. Subsequently, we identified six common genes between the two datasets and validated them in other datasets. Further analysis demonstrated that two genes, including *NR4A2* and *IGFBP1b* with higher AUC, sensitivity, and specificity, were diagnostic biomarkers in fatty liver.

*ABCF1*, also named *ABC50*, is a member of the ABC transporter superfamily protein localized on the cytosol and endoplasmic reticulum (ER), which transport different molecules, including carbohydrates, amino acids, and ions. Furthermore, *ABCF1* is critical in regulating innate immune and inflammatory responses^27,28^. This protein is considered an oncofetal protein significantly expressed in the fetal liver, not healthy adult cells. Fung et al. showed that the expression of *ABCF1* was increased in hepatocellular carcinoma (HCC), and was associated with chemoresistance^29^. Cheung et al. demonstrated that upregulated *ABCF1* gene is associated with poor recurrence-free survival (RFS) in liver cancer^30^. A significant association between other members of the ABC family and NAFLD has been proven in previous studies. *ABCB1* plays a crucial role in transporting phospholipids and cholesterol into the liver cells. An animal study exhibited that the level of transporter proteins such as *ABCB1, ABCC1-6*, and *ABCG2* increased during the progression of NASH^31^. The *ABCB1* is overexpressed in liver diseases such as cholestatic, biliary cirrhosis, and obstructive jaundice^32–34^. The *SART3* and *RNPS1* are the genes with the highest score in the advanced stage of NAFLD; moreover, the result of PPI revealed that there is a strong correlation between *SART3* and *RNPS1*, both of them are members of the post-splicing complex. *SART3* is known as tumor-associated antigens detected in HCC and makes hepatocytes sensitive to immunotherapy^35^. A previous study used two datasets of GEO (GSE33814 and GSE89632) and showed that *RNPS1* is one of the top genes overexpressed in NAFLD cells compared to the control group. *RNPS1* is a member of the post-splicing complex role in RNA processing and apoptosis^36^. One of the other key genes detected in our investigation was *APC5*, a subunit of the anaphase-promoting complex (APC). Zhang et al. showed *APC5* plays a critical role in activating the cell cycle during adipose tissue proliferation^37^. A study showed that after feeding, the expression of *NONO* gene significantly increased to uptake glucose. Furthermore, the results revealed that the deficient-*NONO* gene in mice reduces triglyceride storage and increases hepatocyte lipid catabolism^38^. In a current study, Wu et al. indicated that the expression of *NONO* gene was highly elevated in NAFLD mice^39^. our result indicates that *CNTNAP1* is upregulated in NAFLD, which agrees with the previous study. *CNTNAP1* has a positive role in triglyceride metabolism^40^. *KAT7* gene, also known as *HBO1*, belongs to the lysine acetyltransferase family, which is a key factor in forming a replication complex, regulating the immune system and developing embryonic development. Information confirmed that the expression of *KAT7* in mRNA and protein levels elevated in HCC cells leads to the proliferation and invasion of tumor cells. Zhong et al. reported that silencing the *KAT7* gene using short hairpin RNA (shRNA) and CRISPR/Cas9 in the xenograft HCC model inhibited tumorigenesis^41^. *ZPR1* is a zinc finger family member, and Wo et al. showed patients with severe NAFLD had *ZPR1* rs964184 polymorphism. we hypothesized that this polymorphism could be associated with high expression of *ZPR1* in patients^42^.

The analysis of the GSE126848 dataset revealed that the expression of *RABGAP1* gene *is* associated with NAFLD***.*** The previous studies showed Rabgap1 expression raised in perirenal fat and brown fat in *Gpr21* knockout mice when fed with a high-fat diet^43^. Rabgap1 GTPase Activating protein which transited the cells from metaphase to anaphase. *SLC7A8* and *SPAG9* are two novel DEGs identified in our study. *SLC7A8*, the light-chain subunit solute carrier family 7, member 8, is a vital gene in inducing hypertrophy in adipose tissue and inflammation. Pitere et al. reported that the SLC7A8 deficiency in mice with diet-induced obesity decreases lipid accumulation in the liver^44^. *SPAG9* is expressed explicitly in the testis and has a vital role in fertility. A study on chicken illustrated that the samples that overexpressed the *SPAG9* gene have more fat content on the abdominal and liver tissues^45^. Furthermore, *SPAG9* increases the proliferation of HCC cells through the interaction with MAPK/Jun pathway^45^. *KAT6A* is another member of the lysine acetyltransferase family, which epigenetically regulates the transcription of different genes involved in DNA repairing systems, cell cycle, metabolism, and autophagy. Many studies confirmed the overexpression of *KAT6A* related to HCC progression and chemoresistance^46,47^.

Our result revealed a significant relationship between clinical and demographic data, including fat, fibrosis, body mass index (BMI), inflammation, and fatty liver. In many studies, BMI is announced as a critical index for increasing the risk of fatty liver. The BMI score of patients is a 4 to 14-fold change higher than healthy individuals. Fan et al. reported that 73% of patients with NAFLD were obese and overweight^48^. BMI measurement is a helpful and non-invasive marker for predicting fatty liver. They suggested triple approaches comprising examining the lipid panel, BMI measurement, and radiological techniques^49,50^. Inflammation and fibrosis are the major pathological consequences of NAFLD. Fibrogenesis is stimulated by the activation of hepatic stellate cells and Kupffer cells, resulting from high plasma levels of glucose and lipids^51^. The activated hepatic stellate cells express different myogenic and pro-inflammatory markers such as myocyte enhancer factor-2 (*Mef2*), *c-myb*, and *TGF-β.* Moreover, inflammation results from increasing the level of reactive oxygen species (ROS) and cytokines in liver tissue^52,53^. The result of a meta-analysis revealed that the fibrosis stage significantly correlates with the risk of mortality in NAFLD^54^.

We reported NR4A2 and IGFBP1b as novel diagnostic biomarkers in fatty liver. Insulin-like growth factor binding protein (IGFBP) binds to insulin-like growth factors (IGFs) and regulates cellular metabolism. Hepatocytes largely produce IGFBP and secrete it into the serum. Previous studies are in line with our results, Pan et al. reported a high expression of IGFBP in NAFLD patients, L02 cells, and also in mice models of NAFLD^55^. NR4A2 is a transcription factor that plays a pivotal role in regulating fatty acid beta-oxidation. Therefore, the dysregulation of NR4A2 causes fat accumulation in the liver^56^. Chen et al. showed that NR4A2 overexpression prevents Hepatic stellate cell (HSCs) proliferation which plays a key role in liver fibrogenesis^57^.

Previous evidence confirmed that novel approaches, including machine learning, are promising strategies for diagnosing, preventing, and managing diseases. Wu et al. compared four machine learning algorithms in predicting fatty liver disease, and they showed that the random forest model has a higher performance in the early diagnosis of fatty liver^58^. The result of a cross-sectional investigation showed that machine learning is a predictive model of NAFLD. They revealed that this method enhances clinical decisions and reduces end-stage disease^59^. Furthermore, previous studies used machine learning methods for identifying novel biomarkers in various conditions, such as cancer^60–62^, cardiovascular diseases^63,64^, pulmonary diseases^65,66^, and neurological disorders^67,68^.

In conclusion, using a bioinformatic approach; twelve key genes were detected that are significantly related to the fatty liver. It is recommended that these key genes are assessed further as possible predictive markers during the development of the fatty liver.

### Supplementary Information

Supplementary Information.

## Data Availability

The datasets generated and/or analysed during the current study are available in the GEO repository, https://www.ncbi.nlm.nih.gov/geo/geo2r/?acc=GSE48452 and https://www.ncbi.nlm.nih.gov/geo/query/acc.cgi?acc=GSE126848.
