# Supplementary material for: Bioinformatics analysis and machine learning approach applied to the identification of novel key genes involved in non-alcoholic fatty liver disease
Source: Sci Rep. 2023 Nov 22;13:20489. doi: 10.1038/s41598-023-46711-x (PMC10665370; doi:10.1038/s41598-023-46711-x)
Supplement: Supplementary file 1 — Supplementary Information. [file 41598_2023_46711_MOESM1_ESM.docx]

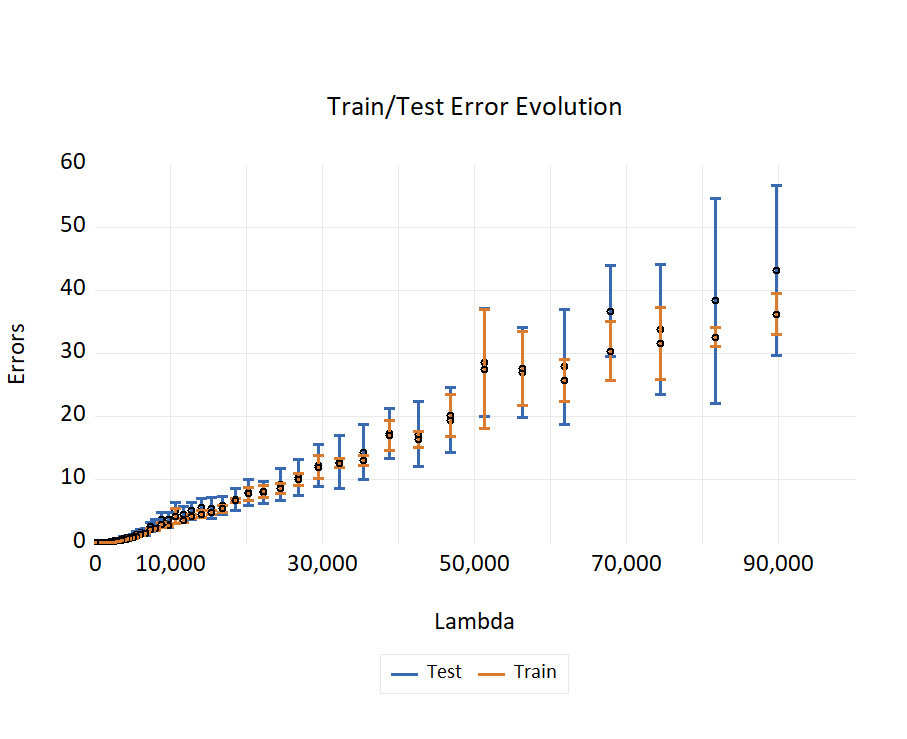


Figure S1. Train/Test Error for different values of Lambda in the Elastic Net model


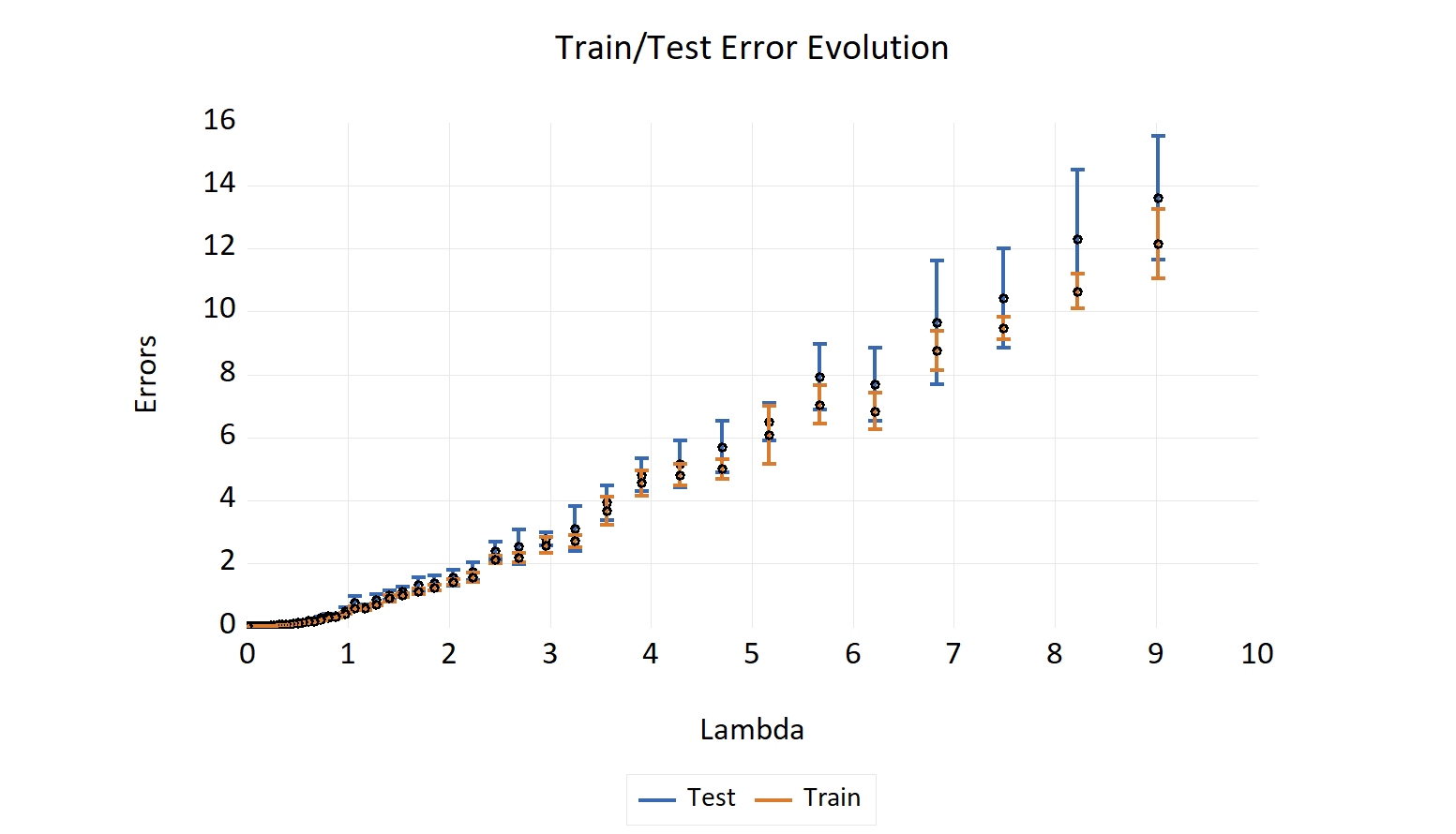
 Figure S2. Train/Test Error for different values of Lambda in the Elastic Net model


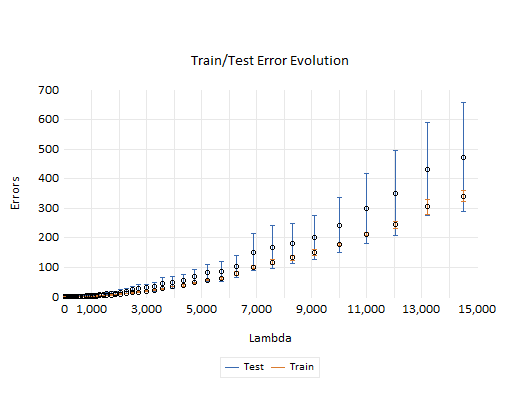


Figure S3. Train/Test Error for different values of Lambda in the Lasso model (GSE126848)


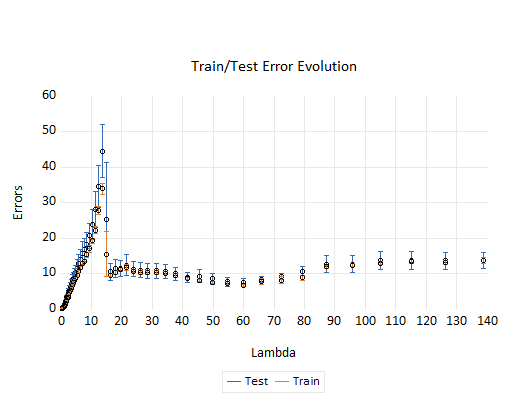


Figure S4. Train/Test Error for different values of Lambda in the Lasso model (GSE48452)


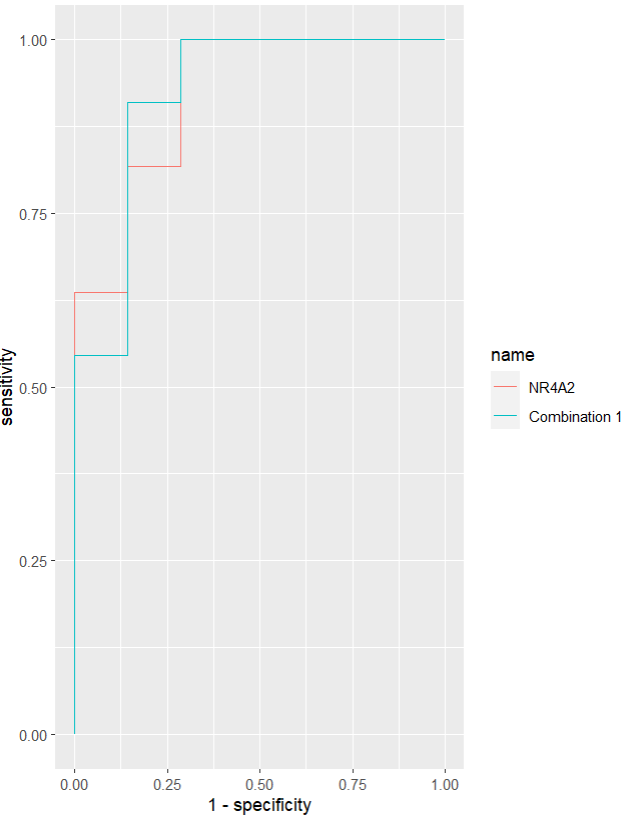


Figure S5: ROC cure of NR4A2 gene and its combination with ZEB2

**Table S1:** Results of the diagnostic tests performed between the fatty liver patients and the controls for NR4A2 gene and its combination with ZEB2

|  | AUC | SE | SP | CutOff | ACC | TN | TP | FN | FP | NPV | PPV |
| --- | --- | --- | --- | --- | --- | --- | --- | --- | --- | --- | --- |
| NR4A2 | 0.922 | 1.000 | 0.714 | 0.208 | 0.889 | 5 | 11 | 0 | 2 | 1.000 | 0.846 |
| Combination | 0.922 | 0.909 | 0.857 | 0.538 | 0.889 | 6 | 10 | 1 | 1 | 0.857 | 0.909 |

| Diagnosis Biomarkers and their combinations | Intercept | Coefficients | Degrees of Freedom | Null Deviance | Residual Deviance | AIC |
| --- | --- | --- | --- | --- | --- | --- |
| NR4A2 | 361.0 | log(NR4A2 + 1) : -193.9 | 17 | 24.06 | 12.35 | 16.35 |
| Combination 1(NR4A2-ZEB2) | 369.206 | log(NR4A2 + 1): -194.223  log(ZEB2 + 1) : -4.326 | 17 | 24.06 | 12.34 | 18.34 |


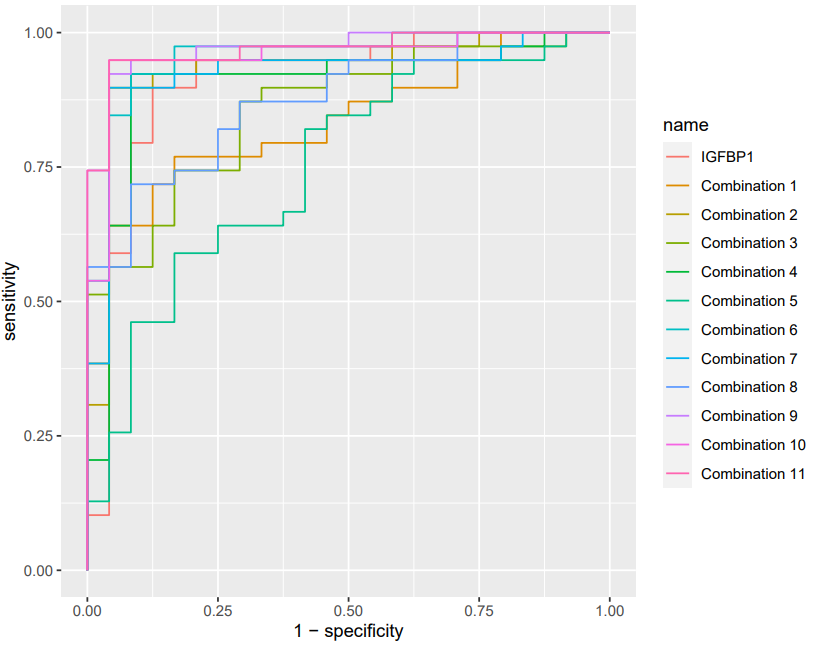


**Figure S6:** ROC cure of IGFBP1b gene and its combination with other genes

Table S2: Results of the diagnostic tests performed between the fatty liver patients and the controls for IGFBP1b gene and its combination

|  | AUC | SE | SP | CutOff | ACC | TN | TP | FN | FP | NPV | PPV |
| --- | --- | --- | --- | --- | --- | --- | --- | --- | --- | --- | --- |
| IGFBP1 | 0.909 | 0.897 | 0.875 | 0.458 | 0.889 | 21 | 35 | 4 | 3 | 0.840 | 0.921 |
| Combination 1  (AKR1B10-DHRS2) | 0.834 | 0.769 | 0.833 | 0.526 | 0.794 | 20 | 30 | 9 | 4 | 0.690 | 0.882 |
| Combination 2  ) AKR1B10-IGFBP1( | 0.933 | 0.897 | 0.958 | 0.548 | 0.921 | 23 | 35 | 4 | 1 | 0.852 | 0.972 |
| Combination 3  ) AKR1B10-UGT2B17( | 0.859 | 0.872 | 0.708 | 0.490 | 0.810 | 17 | 34 | 5 | 7 | 0.773 | 0.829 |
| Combination 4  ) DHRS2-IGFBP1( | 0.904 | 0.923 | 0.917 | 0.449 | 0.921 | 22 | 36 | 3 | 2 | 0.880 | 0.947 |
| Combination 5  ) DHRS2-UGT2B17( | 0.752 | 0.590 | 0.833 | 0.711 | 0.683 | 20 | 23 | 16 | 4 | 0.556 | 0.852 |
| Combination 6  ) IGFBP1-UGT2B17( | 0.957 | 0.923 | 0.917 | 0.456 | 0.921 | 22 | 36 | 3 | 2 | 0.880 | 0.947 |
| Combination 7  ) AKR1B10-DHRS2-IGFBP1( | 0.926 | 0.897 | 0.958 | 0.526 | 0.921 | 23 | 35 | 4 | 1 | 0.852 | 0.972 |
| Combination 8  ) AKR1B10-DHRS2-UGT2B17( | 0.876 | 0.718 | 0.917 | 0.700 | 0.794 | 22 | 28 | 11 | 2 | 0.667 | 0.933 |
| Combination 9  ) AKR1B10-IGFBP1-UGT2B17( | 0.972 | 0.923 | 0.958 | 0.469 | 0.937 | 23 | 36 | 3 | 1 | 0.885 | 0.973 |
| Combination 10  ) DHRS2-IGFBP1-UGT2B17( | 0.956 | 0.949 | 0.958 | 0.425 | 0.952 | 23 | 37 | 2 | 1 | 0.920 | 0.974 |
| Combination 11  ) AKR1B10-DHRS2-IGFBP1-UGT2B17( | 0.969 | 0.949 | 0.958 | 0.309 | 0.952 | 23 | 37 | 2 | 1 | 0.920 | 0.974 |

| Diagnosis Biomarkers and their combinations | Intercept | Coefficients | Degrees of Freedom | Null Deviance | Residual Deviance | AIC |
| --- | --- | --- | --- | --- | --- | --- |
| IGFBP1 | 63.38 | log(IGFBP1 + 1) : -25.46 | 62 | 83.73 | 51.02 | 55.02 |
| Combination1 | -34.307 | log(AKR1B10 + 1): 10.022  log(DHRS2 + 1) : 4.644 | 62 | 83.73 | 59.98 | 65.98 |
| Combination2 | 34.82 | log(AKR1B10 + 1): 10.21  log(IGFBP1 + 1) : -23.65 | 62 | 83.73 | 38.92 | 44.92 |
| Combination3 | -252.15 | log(AKR1B10 + 1) :10.88  log(UGT2B17 + 1) :82.44 | 62 | 83.73 | 55.5 | 61.5 |
| Combination4 | 47.912 | log(DHRS2 + 1) :6.122  log(IGFBP1 + 1) : -25.080 | 62 | 83.73 | 49.04 | 55.04 |
| Combination5 | -333.786 | log(DHRS2 + 1) : 8.196  log(UGT2B17 + 1) : 114.332 | 62 | 83.73 | 69.98 | 75.98 |
| Combination6 | -323.93 | log(IGFBP1 + 1) : -43.09  log(UGT2B17 + 1) : 156.77 | 62 | 83.73 | 36.71 | 42.71 |
| Combination7 | 25.402 | log(AKR1B10 + 1): 9.502  log(DHRS2 + 1) : 3.860  log(IGFBP1 + 1) : -22.891 | 62 | 83.73 | 38.32 | 46.32 |
| Combination8 | 324.296 | log(AKR1B10 + 1) : 10.114  log(DHRS2 + 1) : 7.193  log(UGT2B17 + 1) : 103.063 | 62 | 83.73 | 52.35 | 60.35 |
| Combination9 | -396.02 | log(AKR1B10 + 1) : 14.91  log(IGFBP1 + 1) : -44.96  log(UGT2B17 + 1) : 171.99 | 62 | 83.73 | 25.51 | 33.51 |
| Combination10 | -340.282 | log(DHRS2 + 1): 6.297  log(IGFBP1 + 1) : -41.710  log(UGT2B17 + 1) : 156.041 | 62 | 83.73 | 35.01 | 43.01 |
| Combination11 | -392.334 | log(AKR1B10 + 1) : 13.837  log(DHRS2 + 1) : 2.463  log(IGFBP1 + 1) : -43.531  log(UGT2B17 + 1) : 168.138 | 62 | 83.73 | 25.33 | 35.33 |
